# Longitudinal SARS-CoV-2 seroepidemiological investigation among healthcare workers at a tertiary care hospital in Germany

**DOI:** 10.1186/s12879-022-07057-3

**Published:** 2022-01-24

**Authors:** Sara Tomczyk, Alexander Hönning, Julia Hermes, Marica Grossegesse, Natalie Hofmann, Janine Michel, Markus Neumann, Andreas Nitsche, Berthold Hoppe, Tim Eckmanns, Hajo Schmidt-Traub, Kristina Zappel

**Affiliations:** 1 Department of Infectious Disease Epidemiology, Robert Koch Institute, Seestrasse 10, 13353 Berlin, Germany; 2 Centre for Clinical Research, BG Klinikum Unfallkrankenhaus Berlin gGmbH, Berlin, Germany; 3 Highly Pathogenic Viruses, Centre for Biological Threats and Special Pathogens, WHO Reference Laboratory for SARS-CoV-2 and WHO Collaborating Centre for Emerging Infections and Biological Threats, Robert Koch Institute, Berlin, Germany; 4 BG Klinikum Unfallkrankenhaus Berlin gGmbH, Berlin, Germany

**Keywords:** SARS-CoV-2, Seropositivity, Health care worker, Outbreak, Tertiary hospital, Germany

## Abstract

**Background:**

SARS-CoV-2 cases in Germany increased in early March 2020. By April 2020, cases among health care workers (HCW) were detected across departments at a tertiary care hospital in Berlin, prompting a longitudinal investigation to assess HCW SARS-CoV-2 serostatus with an improved testing strategy and associated risk factors.

**Methods:**

In May/June and December 2020, HCWs voluntarily provided blood for serology and nasopharyngeal/oropharyngeal (NP/OP) samples for real-time polymerase chain reaction (PCR) and completed a questionnaire. A four-tiered SARS-CoV-2 serological testing strategy including two different enzyme-linked immunosorbent assays (ELISA) and biological neutralization test (NT) was used. ELISA-NT correlation was assessed using Pearson’s correlation coefficient. Sociodemographic and occupational factors associated with seropositivity were assessed with multivariate logistic regression.

**Results:**

In May/June, 18/1477 (1.2%) HCWs were SARS-CoV-2 seropositive, followed by 56/1223 (4.6%) in December. Among those tested in both, all seropositive in May/June remained seropositive by ELISA and positive by NT after 6 months. ELISA ratios correlated well with NT titres in May/June (R = 0.79) but less so in December (R = 0.41). Those seropositive reporting a past SARS-CoV-2 positive PCR result increased from 44.4% in May/June to 85.7% in December. HCWs with higher occupational risk (based on profession and working site), nurses, males, and those self-reporting COVID-19-like symptoms had significantly higher odds of seropositivity.

**Conclusions:**

This investigation provides insight into the burden of HCW infection in this local outbreak context and the antibody dynamics over time with an improved robust testing strategy. It also highlights the continued need for effective infection control measures particularly among HCWs with higher occupational risk.

**Supplementary Information:**

The online version contains supplementary material available at 10.1186/s12879-022-07057-3.

## Background

Following the emergence and rapid global spread of severe acute respiratory syndrome coronavirus-2 (SARS-CoV-2), the World Health Organization (WHO) declared the coronavirus disease 2019 (COVID-19) outbreak a global pandemic on 11 March 2020 [1]. In Germany, the first laboratory-confirmed SARS-CoV-2 case was reported on 27 January 2020 [2]. Cases of SARS-CoV-2 in Germany started to significantly increase in early March 2020 (‘first wave’), with a peak of approximately 35,000 reported cases in calendar week 14, leading to wide-reaching impacts on the German health system. Although cases of SARS-CoV-2 declined in Germany during the following summer months, increasingly diffuse infection chains and outbreak settings were seen. In October 2020, cases of SARS-CoV-2 again started to increase (‘second wave’), and the mean number of occupied intensive care unit (ICU) beds grew from 807 to 5643 in calendar week 13–53 [2].

In many settings, the burden of the COVID-19 pandemic has stressed the capacity of hospitals and ICUs. In Berlin, Germany, a standardised approach was developed to distribute COVID-19 patients requiring invasive ventilation across hospitals in order to optimize the flow and care of these at-risk patients (“SAVE-Model”) [3]. This includes a ‘first-level’ coordinating hospital and 16 ‘second-level’ specialised hospitals with the capacity to treat critically-ill COVID-19 patients. The remaining 60 ‘third-level’ hospitals with emergency services are designated to provide intensive care for non-COVID-19 patients.

However, the capacity of health service delivery and the COVID-19 pandemic response is largely dependent on the availability and protection of health care workers (HCWs). HCWs may have high-risk occupational exposures to SARS-CoV-2 infectious material. Hence, effective infection prevention and control (IPC) measures such as appropriate use of personal protection equipment (PPE) and early detection of SARS-CoV-2 infection among HCWs are critical to prevent and control nosocomial transmission [4–7]. A meta-analysis of 49 studies including 127,480 HCWs in North America, Europe, Africa and Asia estimated an overall seroprevalence of SARS-CoV-2 of 8.7% among HCW [8]. However, studies in Stockholm and London found that HCW seropositivity was considerably higher than that of the general population, although such findings can be closely related to local outbreak dynamics [9, 10].

By 3 April 2020, 25 SARS-CoV-2 HCW cases had been detected across different departments at a tertiary care hospital in Berlin, Germany and reported to the local public health department. An outbreak was declared, and the regional and federal public health service were invited to assist in further response measures. This prompted systematic hospital-wide staff screening and an intensified outbreak investigation. By repeatedly assessing the SARS-CoV-2 serostatus of the HCWs and associated factors, we aimed to elucidate the chain of infection and prevent future transmission of disease in this health care setting.

## Methods

### Hospital

The tertiary care hospital ‘Unfallkrankenhaus Berlin (ukb)’ in Berlin, Germany is a maximum care trauma centre with more than 730 beds, 17 operating theatres, over 2500 HCWs including the trauma treatment centre and subsidiaries serving a catchment area of approximately 300,000 inhabitants. According to the ‘SAVE-Model’ in Berlin (i.e. distribution of COVID-19 patients requiring invasive ventilation), the ukb hospital serves as one of the 16 specialized hospitals with the capacity to treat critically-ill COVID-19 patients [3].

In February–March 2020, a comprehensive SARS-CoV-2 pandemic response plan was developed and implemented at ukb hospital. HCWs were trained on IPC measures, including protocols for enhanced hand hygiene, deep cleaning, PPE, patient cohorting, isolation, testing and self-monitoring of symptoms. Upon admission, COVID-19 confirmed and suspected cases were spatially separated and tested for SARS-CoV-2. COVID-19 cases were then treated on designated wards and segregation of staff on these wards was encouraged. The first cases of COVID-19 were admitted to ukb hospital on 9 March 2020. After SARS-CoV-2 infections among HCWs occurred, staff were closely monitored including routine testing all symptomatic HCWs. Until February 2021, the ukb hospital cared for 582 patients with COVID-19, including 232 (39.9%) on intensive care units.

### Outbreak investigation

**Fig. 1:**
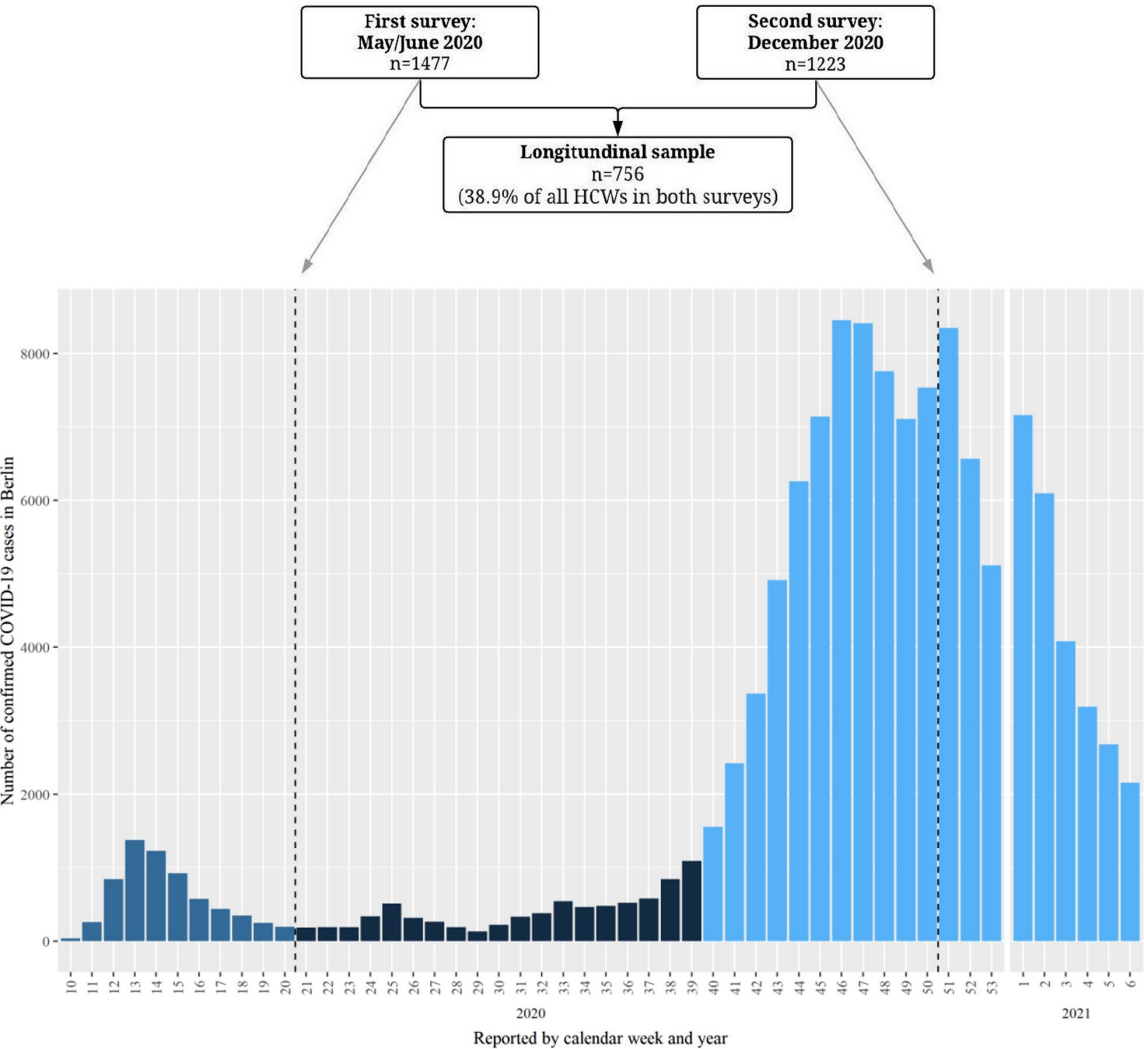
Hospital investigation in the context of the COVID-19 outbreak in Berlin, Germany (N = 1944). *Dark blue denotes the first COVID-19 wave and light blue denotes the second COVID-19 wave

We asked all HCWs to participate in a first survey from 18 May to 10 June 2020 (following the ‘first wave’ of the COVID-19 outbreak from calendar week 10–20; Fig. 1) [2]. HCWs included clinical personnel as well as personnel without direct patient contact. Participants who provided written informed consent were asked to complete a paper questionnaire to collect data on sociodemographics, occupational and community exposures, use of infection prevention and control (IPC) measures, and SARS-CoV-2 testing (e.g. time polymerase chain reaction [PCR]) and symptom history. A sample of about 5–6 mL of peripheral venous blood was collected from each participant and stored at + 4 °C until laboratory testing. Additionally, a voluntary nasopharyngeal/oropharyngeal (NP/OP) sample was collected (eSwab™, Copan) from those that agreed. Among those testing seropositive, a second blood sample was collected in August 2020.

A second survey was conducted from 8 to 20 December 2020 (during the ‘second wave’ of the COVID-19 from week 40; Fig. 1) [2], and all HCWs were again invited to participate, including a shorter questionnaire and second blood sample. The ‘longitudinal sample’ consisted of HCWs who participated in both the first and second survey investigations.

A formal ethical review process and approval was not required for this outbreak investigation in accordance with article 25, section 1 of the German Protection against Infection Act (IfSG).

### Laboratory procedures

Serological analyses for SARS-CoV-2 were performed using a four-tiered testing strategy (Additional file 1: sections 1 and 2). In short, in step one, samples were screened with the semiquantitative Euroimmun SARS-CoV-2 Immunoglobulin G (IgG) enzyme-linked immunosorbent assay (ELISA) with S1 domain substrate (Euroimmun AG, Lübeck, Germany); in step two, repeat testing was conducted with the Euroimmun IgG ELISA in duplicate; and in step three, the WANTAI SARS-CoV-2 Ab ELISA (Beijing Wantai Biological Pharmacy Enterprise; Beijing, China) was used as a verification assay. In step four, if Euroimmun and Wantai ELISA results were discordant, an in-house biological neutralization test (NT) was performed (further details in Additional file 1: section 1). With the NP/OP swabs from the first survey, real-time PCR was performed as previously published by Michel et al. [11].

### Case definition

A seropositive case was defined as those with either a (1) positive or borderline result by Euroimmun and confirmed positive by Wantai, or (2) positive or borderline result by Euroimmun, discordant results by Wantai but presence of neutralizing antibodies by the biological NT.

### Statistical analysis

Missing questionnaire data were not imputed but presented for each variable. A variable for SARS-CoV-2 occupational risk was created based on type of profession and working site as previously described [12]. If a HCW could not exclusively be classified into one category, she/he was assigned to the higher risk category. The categories were defined as follows:

1. High risk: personnel working on separate COVID-19-dedicated general wards and ICUs; Job functions included physicians, nurses, other allied health professionals (e.g. medical-technical assistants, therapists, psychologists, and social workers) and cleaning/catering staff.
2. Moderate risk: personnel working on wards which may have a higher risk for SARS-CoV-2 transmission if a patient had COVID-19, including anaesthesia, Ear-Nose-Throat departments, Oral and Maxillofacial Surgery departments, and non-COVID-19 ICUs; Same job functions as above.
3. Low risk: personnel having direct contact to patients or samples outside of high and moderate risk categories; Same job functions as above in addition to laboratory staff.
4. Very low risk: personnel having no direct contact to patients; Job functions included administrative staff and facility management without patient contact.

Estimated SARS-CoV-2 seroprevalence was assessed according to survey time period and factors collected in the questionnaire with absolute and relative frequencies. A sensitivity analysis was conducted to compare the final results using the four-tiered testing strategy with results using only the Euroimmun ELISA ratio adjusted for test performance (Additional file 1: section 3). Among those tested with both assays, the correlation of the SARS-CoV-2 Euroimmun IgG ELISA ratios and the NT titres was assessed graphically and using the Pearson’s correlation coefficient (*R*).

Binary and multivariate logistic regression was conducted to further evaluate the association of SARS-CoV-2 seropositivity and factors from the second survey. Two separate multivariate models were used to assess collider bias: (1) fully adjusted (adjusted for all selected covariates) and (2) mutually adjusted (adjusted only for occupational risk categories and type of profession). Discordant model results could indicate the causal influence of covariates on occupational risk and type of profession [13]. Multicollinearity was assessed using model diagnostics. EpiData version 2.0 and Excel version 2019 were used for data entry; R version 4.0.5 (R Foundation for Statistical Computing, Vienna, Austria) and SPSS V27.0 (IBM Deutschland GmbH, Ehningen, Germany) were used for all statistical analyses.

## Results

A total of 1944 HCWs participated in the May–June and/or December 2020 cross-sectional surveys (see Fig. 1). Among these, a longitudinal sample of 756 (38.9%) HCWs were included in both surveys. Approximately 70% of participants were women, and the median age was around 40 years. Approximately half were physicians or nurses, and more than 18% of HCWs were determined to have a high or moderate occupational risk to SARS-CoV-2 (Table 1).

**Table 1:** Characteristics of HCW participants by survey sample and SARS-CoV-2 seropositivity

Variable	May/June 2020			December 2020			Longitudinal sample
	All (N = 1477)	Seropositive (N = 18)	Seronegative (N = 1459)	All (N = 1223)	Seropositive (N = 56)	Seronegative (N = 1167)	All (N = 756)
Age in years, median (IQR)	41 (32–51)	37 (27.8–49.3)	41 (32–51)	40 (32–51)	40 (30–47)	40 (32–51)	43 (34–52)
Age groups in years							
16–29	189 (12.8%)	5 (27.8%)	184 (12.6%)	176 (14.4%)	12 (21.4%)	164 (14.0%)	66 (8.7%)
30–39	503 (34.1%)	4 (22.2%)	499 (34.2%)	405 (33.1%)	15 (26.8%)	390 (33.4%)	246 (32.5%)
40–49	349 (23.6%)	5 (27.8%)	344 (23.6%)	284 (23.2%)	20 (35.7%)	264 (22.6%)	195 (25.8%)
50–59	358 (24.2%)	3 (16.7%)	355 (24.3%)	281 (23.0%)	6 (10.7%)	275 (23.5%)	211 (27.9%)
60+	73 (4.9%)	1 (5.6%)	72 (4.9%)	58 (4.7%)	2 (3.6%)	56 (4.8%)	38 (5.0%)
Unknown	5 (0.3%)	0	5 (3.4%)	19 (1.6%)	1 (1.8%)	19 (1.6%)	0 (0.0%)
Gender							
Female	1038 (70.3%)	12 (66.7%)	1026 (70.3%)	842 (68.8%)	31 (55.4%)	811 (69.5%)	527 (69.7%)
Male	436 (29.5%)	6 (33.3%)	430 (29.5%)	380 (31.1%)	24 (42.9%)	356 (30.5%)	229 (30.3%)
Diverse	1 (0.1%)	0	1 (0.1%)	1 (0.1%)	1 (1.8%)	0	0 (0.0%)
Unknown	2 (0.1%)	0	2 (0.1%)	0 (0.0%)	0	0	0 (0.0%)
Self-reported COVID-19-like symptoms^a^							
Yes	140 (9.5%)	7 (38.9%)	133 (9.1%)	193 (15.8%)	21 (37.5%)	172 (14.7%)	115 (15.2%)
No	1148 (77.8%)	10 (55.6%)	1138 (78.0%)	1013 (82.8%)	34 (60.7%)	979 (83.9%)	627 (82.9%)
Unknown	189 (12.8%)	1 (5.6%)	188 (12.9%)	17 (1.4%)	1 (1.8%)	16 (13.7%)	14 (1.9%)
Type of profession							
Nurse	469 (31.8%)	6 (33.3%)	463 (31.7%)	326 (26.7%)	22 (39.3%)	305 (26.1%)	189 (25.0%)
Physician	307 (20.8%)	8 (44.4%)	299 (20.5%)	264 (21.6%)	14 (25.0%)	250 (21.4%)	167 (22.1%)
Other allied health professionals	298 (20.2%)	2 (11.1%)	296 (20.3%)	276 (22.6%)	10 (17.9%)	266 (22.8%)	178 (23.5%)
Administration/other facility management	367 (24.8%)	1 (5.6%)	366 (25.1%)	325 (26.6%)	9 (16.1%)	316 (27.1%)	211 (27.9%)
Unknown	37 (2.5%)	1 (5.6%)	35 (2.4%)	31 (2.5%)	1 (1.8%)	30 (2.6%)	11 (1.5%)
SARS-CoV-2 occupational risk							
High	74 (5.0%)	3 (16.7%)	71 (4.9%)	58 (4.7%)	13 (23.2%)	45 (3.9%)	37 (4.9%)
Moderate	204 (13.8%)	8 (44.4%)	196 (13.4%)	200 (16.4%)	12 (21.4%)	188 (16.1%)	99 (13.1%)
Low	843 (57.1%)	7 (38.9%)	836 (57.3%)	619 (50.6%)	26 (46.4%)	593 (50.8%)	394 (52.1%)
Very low	337 (22.8%)	0	337 (23.1%)	332 (27.1%)	5 (8.9%)	327 (28.0%)	218 (28.8%)
Unknown	19 (1.3%)	0	19 (1.3%)	14 (1.1%)	0	14 (1.2%)	8 (1.1%)

*IQR* interquartile range

^a^In May/June, HCWs reported symptoms in the past month; In December, HCWs reported symptoms in the last 14 days

### Estimates of SARS-CoV-2 seropositivity

**Fig. 2:**
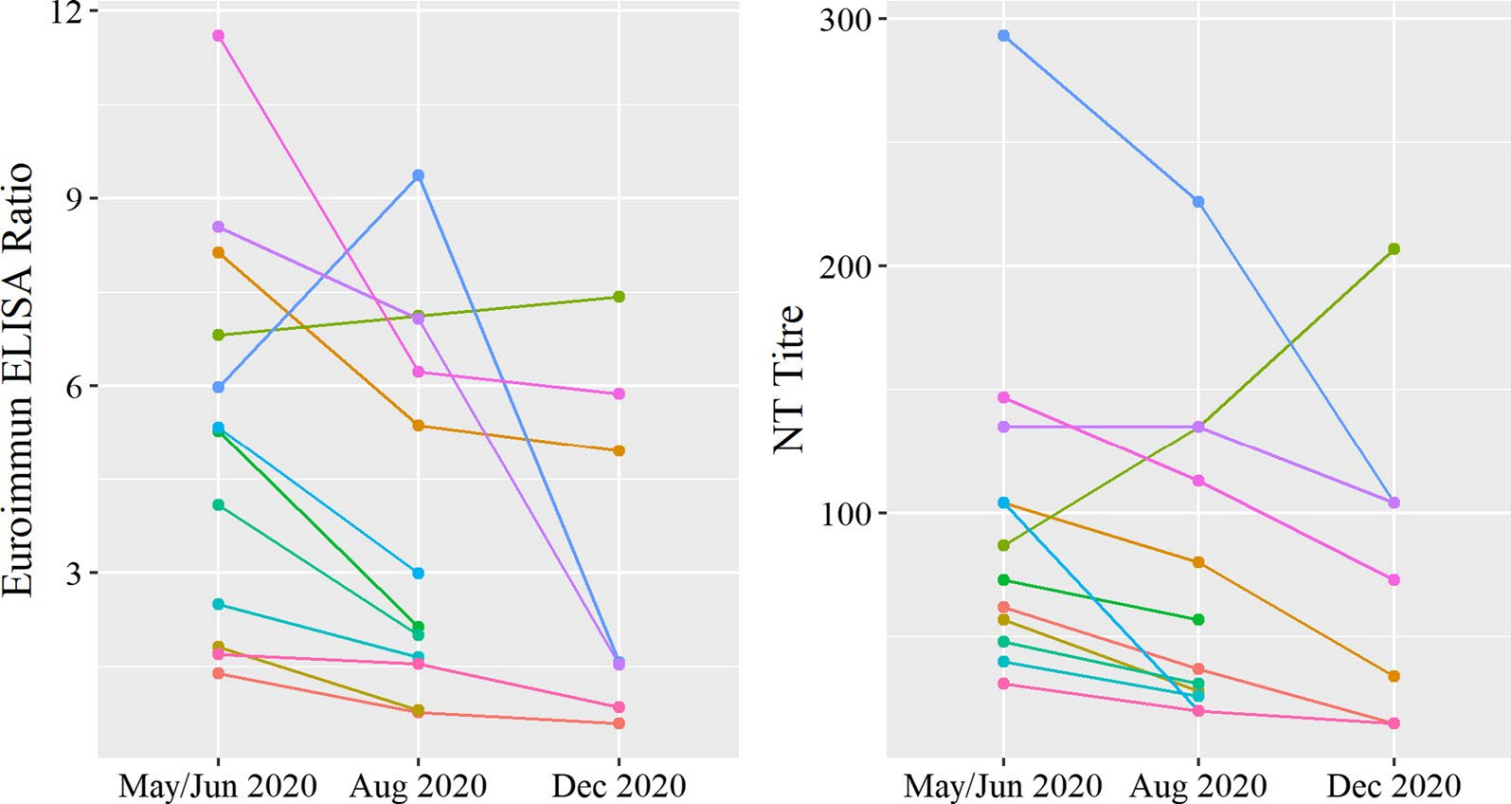
Euroimmun ELISA ratios and NT titres over time among those seropositive in May/June 2020 (N = 12)*. *The results displayed are among those with follow-up testing including seven HCWs from the longitudinal sample with three blood samples between May/June 2020 and December 2020 and five HCW with only a second blood sample in August 2020; The respective colour in both figures corresponds to the same health care worker

**Fig. 3:**
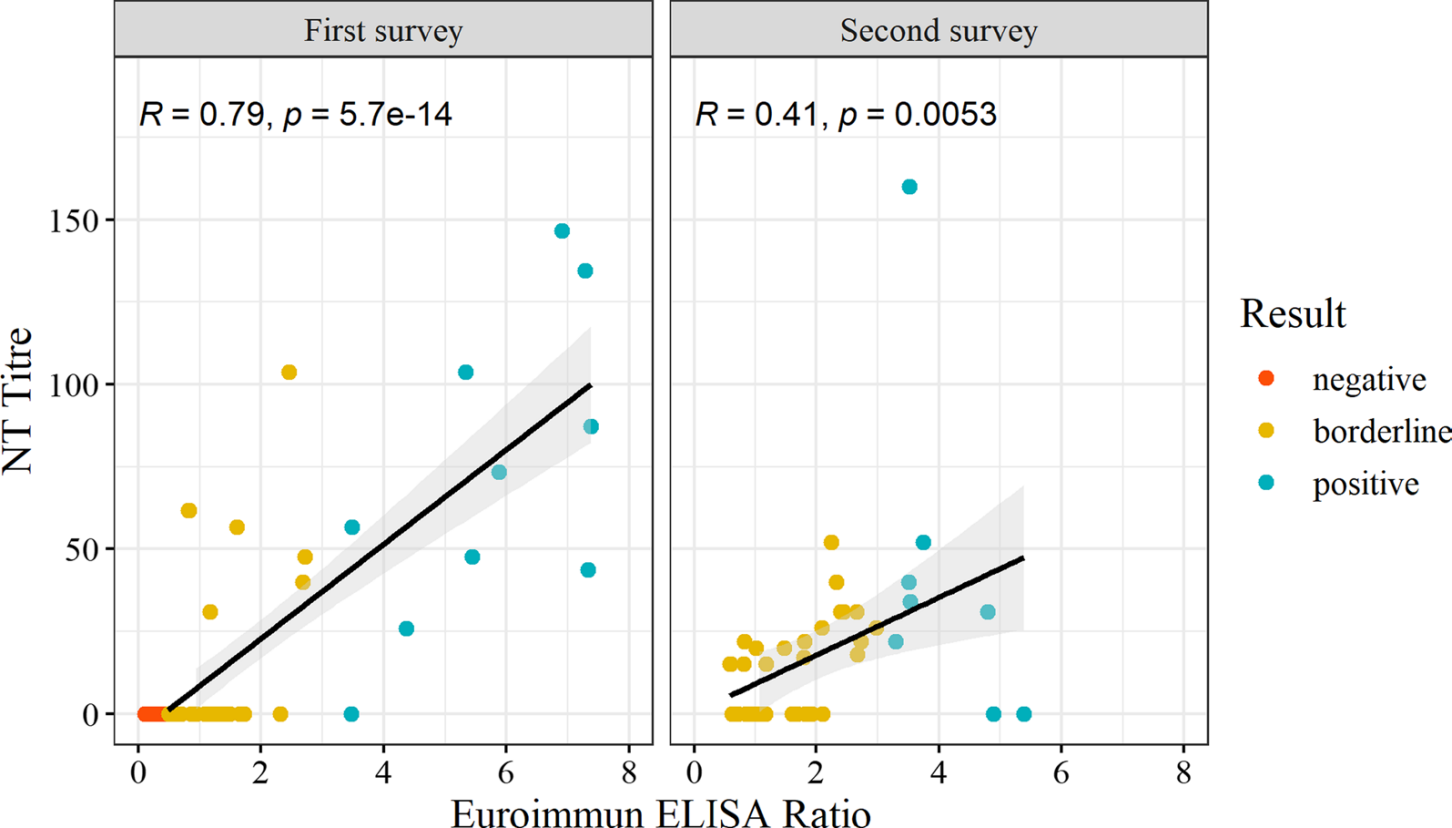
Correlation of Euroimmun ELISA ratios and NT titres among those tested with both assays (N = 122)

In May/June 2020, 18 (1.2%) HCWs were SARS-CoV-2 seropositive, increasing to 56 (4.6%) in December 2020. In the longitudinal sample, seven (0.9%) were seropositive in May/June and 35 (4.6%) in December. All seven HCWs seropositive in the first survey remained seropositive in the follow-up 6 months later. They also remained NT-positive, including one with an increasing titre (Fig. 2). In a sensitivity analysis, the final estimates of seropositivity using the four-tiered testing strategy were not substantially different compared to the results of the Euroimmun ELISA ratios adjusted for test performance in the second survey (Additional file 1: section 3). Euroimmun ELISA ratios correlated well with the NT titres in the first survey (R = 0.79, p < 0.001) compared to the second survey where the correlation was lower but still significant (R = 0.41, p = 0.005) (Fig. 3).

### PCR testing and symptom history

In the first survey, 387 (26%) HCWs agreed to provide a NP/OP swab for SARS-CoV-2 PCR testing. Following the cases among HCWs detected in March and April 2020 in a hospital-wide staff screening, only one (0.3%) was confirmed to be PCR positive in the May/June 2020 survey; this HCW was seropositive in the first and second survey. Moreover, in the questionnaire, 8/18 (44.4%) seropositive HCWs self-reported a past PCR positive result during the first survey, compared to 4/1459 (0.3%) seronegative HCWs. This increased to 48/56 (85.7%) seropositive compared to 18/1167 (1.5%) seronegative HCWs in the second survey, respectively (Additional file 1: section 4). Furthermore, more than half of seropositive tested HCWs did not report COVID-19-like symptoms in the first (55.6%) and second survey (60.7%; Table 1).

### Factors associated with SARS-CoV-2 seropositivity

At the time of the second survey, age of the HCWs was not associated with SARS-CoV-2 seropositivity. However, male HCWs had 2.0 times the odds of being seropositive compared to females in the fully adjusted model (p = 0.021; Table 2). Likewise, HCWs who self-reported COVID-19-like symptoms in the 14 days prior had 3.5 times the odds of being seropositive compared to those who did not report such symptoms in the fully adjusted model (p < 0.001; Table 2). Among those SARS-CoV-2 seropositive, 38.9% and 37.5% reported COVID-19-like symptoms prior to the first and second survey, respectively (Table 1). In the second survey, the most common reported symptoms were cough, rhinorrhoea and loss of smell/taste (Additional file 1: section 5).

**Table 2:** Logistic regression analysis for association between seropositivity and exposure to SARS-CoV-2—December 2020 (N = 1223)

Covariates	Unadjusted		Fully adjusted*		Mutually adjusted*	
	OR	95% CI	OR	95% CI	OR	95% CI
Age group in years						
16–29	2.0	0.4–9.4	1.6	0.3–7.4		
30–39	1.1	0.2–4.8	0.9	0.2–3.9		
40–49	2.1	0.5–9.3	1.6	0.3–7.1		
50–59	0.6	0.1–3.1	0.6	0.1–3.1		
60+	Reference	–	–	–		
Gender						
Male	**1.8**	**1.0–3.0**	**2.0**	**1.1–3.4**		
Female	Reference	–	–	–		
Self-reported COVID-19-like symptoms last 14 days						
Yes	**3.5**	**2.0–6.2**	**3.5**	**2.0–6.4**		
No	Reference	–	–	–		
Type of profession						
Nurse	**2.5**	**1.1–5.6**	**2.7**	**1.1–6.2**	0.659	0.2–1.8
Physician	2.0	0.8–4.6	1.6	0.643–4.2	0.793	0.3–2.3
Other allied health professionals	1.3	0.5–3.3	1.2	0.449–3.2	0.759	0.3–2.2
Administration/other facility management	Reference	–	–	–	–	–
SARS-CoV-2 occupational risk						
High	**18.9**	**6.4–55.5**	**18.5**	**6.0–57.2**	**25.5**	**6.8–95.1**
Moderate	**4.2**	**1.4–12.0**	**3.9**	**1.3–11.6**	**5.6**	**1.5–20.9**
Low	**2.9**	**1.1–7.5**	**2.9**	**1.1–7.7**	**3.5**	**1.1–11.2**
Very low	Reference	–	–	–	–	–

Bold represents a p-value of < 0.05

*OR* odds ratio, *CI* confidence intervals

*The fully adjusted model is adjusted for all covariates in the Table and the mutually adjusted model is adjusted only for occupational risk group and profession. Discordant results between these models could indicate the causal influence of covariates on occupational risk and type of profession

Both the fully adjusted and mutually adjusted models showed a clear association between SARS-CoV-2 occupational risk and seropositivity in the second survey (Table 2). For example, in the fully adjusted model, the largest odds ratios (ORs) were found in the high-risk group (18.5, 95% confidence intervals (CI): 6.0–57.2, p < 0.001), followed by the moderate risk group (3.9, 95% CI 1.3–11.6, p = 0.013), and low risk group (2.9, 95% CI 1.1–7.7, p = 0.037) compared to the reference category of very low risk. Among professions, nurses, in particular, were found to have significantly higher odds of seropositivity (Table 2). Likewise, in the first survey, no seropositive cases were found among those in the very low risk groups compared to 4.1% seropositive in the high-risk group and 3.9% in the moderate risk group (Additional file 1: section 6).

In the first survey in May 2020, HCWs also reported other community and occupational exposures and IPC measures (Additional file 1: section 7). According to this subset, a majority of both seropositive (8/12; 66.7%) and seronegative (319/379; 84.2%) HCWs did not yet report close contact to a confirmed case of COVID-19 outside of the hospital in the 14 days prior, but 50.0% of seropositive and 37.7% of seronegative HCWs reported treating confirmed COVID-19 patients. In the 14 days prior, 6 (50.0%) seropositive and 170 (44.9%) seronegative HCWs reported close contact to colleagues without a medical mask on ≥ 5 days. During patient care, most HCWs reported adequate use of PPE and hand hygiene, particularly during aerosol-generating procedures (Additional file 1: section 7).

## Discussion

We present a repeated seroepidemiological investigation at a large tertiary care hospital in the local context of the COVID-19 outbreak in Berlin, Germany. A robust tiered-testing strategy was applied to assess the presence of antibodies against SARS-CoV-2. Seropositivity estimates were found to be lower than other HCW studies internationally, although all those in the longitudinal sample who tested seropositive in the first survey remained seropositive by ELISA and NT in the follow-up 6 months later. In the risk factor analysis, seropositivity was found to be associated with increasing occupational risk and male gender.

Seroprevalence estimates of 1.2% in May/June 2020 and 4.6% in December 2020 found in our investigation were lower than the meta-analysis by Galanis et al. reporting an overall pooled prevalence of 8.7% among all HCWs globally as well as 8.5% among HCWs in Europe. In contrast, other HCW studies in Germany have found similar estimates to our study, ranging from 1.6 to 5.1% across varying regions, HCW populations and time periods from March to July 2020 [14–20]. Korth et al. found a similar increase in seroprevalence from 2.2% in March–May 2020 to 5.1% in October–December 2020 at a tertiary facility in Western Germany [17]. Lower estimates in Germany could reflect differences in existing health care system infrastructure and implementation of IPC measures as well as a lower SARS-CoV-2 incidence in the population, particularly in the ‘first wave’, compared to other countries. A population-based study in Berlin, Germany, estimated a similar seroprevalence of 4.4% in the community in November–December 2020 [21]. Differences across serosurveys could also be due to varying performance characteristics of serological assays with different targets as highlighted by the utility evaluation of serological assays by Heffernan et al. [22].

A systematic review by Post et al. reported that SARS-CoV-2 antibody dynamics have been well described in the acute phase but there remains limited evidence on longer-term patterns. The review found that IgG peaked at weeks 3–7 post-symptom onset and persisted for at least 8 weeks [23]. We demonstrated that all seven participants in our longitudinal sample who tested seropositive in the first survey remained seropositive by ELISA and NT in the follow-up 6 months later. Although those who self-reported COVID-19-like symptoms in the last 14 days had significantly higher odds of being seropositive, we could not determine the exact rate of seroconversion in asymptomatic versus symptomatic cases since not all participants provided a NP/OP swab for PCR testing. The proportion of seropositive HCWs self-reporting a past positive PCR test result increased from 44.4% in the first survey to 85.7% in the second survey, which could reflect the increase in availability and access to staff screening and testing from the first to second COVID-19 wave.

The Euroimmun ELISA ratios were well correlated with the NT titres in the first survey but less correlated in the second survey. Such findings could be explained by the use of different Euroimmun test lots in each survey, although not all samples were tested by NT which could have biased results. An in-house validation study found that the level of the ratio values obtained in the Euroimmun test lots varied from April to September 2020 [24]. We aimed to address this by applying a tiered testing algorithm with a lower Euroimmun test cut-off followed by a verification assay and NT. Our sensitivity analysis found no substantial differences between our results and those of only the Euroimmun ELISA ratios adjusted for test performance. However, validated lot test performance data is only available for some of the lots (Additional file 1: section 3). Furthermore, the kinetics of the antibody response over time and the difference between the timeframe from infection before the first survey (February–May 2020) and second survey (February–December 2020) may have also affected the correlation of the ELISA ratios and NT titres at the different surveys [25].

In the risk factor analysis, we found that increasing occupational risk based on working site and profession was clearly associated with higher odds of SARS-CoV-2 seropositivity. This is consistent with other HCW studies which have shown an association between SARS-CoV-2 seropositivity and working in COVID-19 wards, ICUs and/or as frontline HCWs [9, 10, 26, 27]. This is likely multifactorial due to hospital versus community exposures, varying implementation of IPC and PPE measures, and differences in behaviour or awareness. In the first survey, few HCWs reported close contact to a confirmed case of COVID-19 outside of the hospital and most reported adequate use of PPE and hand hygiene. However, a little less than half reported close contact to colleagues without a medical mask outside of patient care. This may have contributed to transmission but was only assessed in the first survey subset and these practices likely changed during the course of the pandemic response due to hospital-wide mandatory masking policies. We also found that males had 2 times the odds of SARS-CoV-2 seropositivity. This finding varies across HCW studies [26, 28–30]. It may be due to differences in behaviour or other biological factors, as a higher seropositivity (4.9%) was also found among males compared to females (3.8%) in the population-based study in Berlin, Germany [21].

Several limitations should be considered. Participation was voluntary, so this may have introduced bias in the study sample. However, more than half of HCWs participated in the study and a balanced distribution of type of profession and working site was included. If staff were on sick leave during the two surveys, they would not have been included in the study, although COVID-19 related sick leave was not noted on the days of the surveys. It is unknown what proportion of HCWs who were infected did not mount a detectable antibody response or in whom it had waned by the time of testing. In the first survey, the number of seropositive HCWs was small so the significance of associated factors could not be reliably assessed. Overall, questionnaire responses could have been affected by recall and social desirability biases.

## Conclusion

In conclusion, this investigation provides insight into the burden of infection among HCWs in the context of the local COVID-19 outbreak in Berlin, Germany. Findings highlight the need for effective IPC measures particularly among HCWs with the highest occupational risk to minimize SARS-CoV-2 infection in the present COVID-19 wave. The investigation also adds to the body of evidence on SARS-CoV-2 antibody dynamics over time and provides further reflection on serology testing strategy considerations. Such testing considerations continue to be important for ongoing serosurveys in the current COVID-19 pandemic in order to continue to assess duration of immunity as well as vaccine responses according to risk groups, type of vaccine, and SARS-CoV-2 variants of concern. This is true, for example in Germany, whose outbreak situation remains dynamic including a vaccination coverage of approximately 69% (as of 2 December 2021) and rapid detection of the latest variant of concern Omicron following its designation by WHO in the week prior [31].

## Supplementary Information

**Additional file 1.** 1. Laboratory procedures. 2. Figure. Four-tiered testing strategy for the detection of SARS-CoV-2 antibodies. 3. Table. Stratified seroprevalence sensitivity analysis. 4. Table. Results of SARS-CoV-2 seropositivity compared to reported past PCR positivity in each survey sample. 5. Table. COVID-19-like symptoms, December 2020. 6. Table. SARS-CoV-2 Seroprevalence by characteristics of HCW participants in each survey sample. 7. Table. Self-reported COVID-19 exposures and infection prevention and control (IPC) measures, May 2020. 8. References.

## Data Availability

The datasets used and/or analysed during the current study are available from the corresponding author on reasonable request.

## Abbreviations

CI: Confidence intervals
COVID-19: Coronavirus disease 2019
ELISA: Enzyme-linked immunosorbent assay
IfSG: German Protection against Infection Act
HCWs: Health care workers
IgG: Immunoglobulin G
IPC: Infection prevention and control
ICU: Intensive care unit
NP/OP: Nasopharyngeal/oropharyngeal
NT: Neutralization test
OR: Odds ratio
PCR: Polymerase-chain reaction
PPE: Personal protection equipment
SARS-CoV-2: Severe acute respiratory syndrome coronavirus-2
WHO: World Health Organization
