# Supplementary material for: Longitudinal SARS-CoV-2 seroepidemiological investigation among healthcare workers at a tertiary care hospital in Germany
Source: BMC Infect Dis. 2022 Jan 24;22:80. doi: 10.1186/s12879-022-07057-3 (PMC8784861; doi:10.1186/s12879-022-07057-3)
Supplement: Supplementary file 1 — Additional file 1. 1. Laboratory procedures. 2. Figure. Four-tiered testing strategy for the detection of SARS-CoV-2 antibodies. 3. Table. Stratified seroprevalence sensitivity analysis. 4. Table. Results of SARS-CoV-2 seropositivity compared to reported past PCR positivity in each survey sample. 5. Table. COVID-19-like symptoms, December 2020. 6. Table. SARS-CoV-2 Seroprevalence by characteristics of HCW participants in each survey sample. 7. Table. Self-reported COVID-19 exposures and infection prevention and control (IPC) measures, May 2020. 8. References. [file 12879_2022_7057_MOESM1_ESM.docx]

**Additional files**

1. Laboratory procedures
2. Figure. Four-tiered testing strategy for the detection of SARS-CoV-2 antibodies
3. Table. Stratified seroprevalence sensitivity analysis
4. Table. Results of SARS-CoV-2 seropositivity compared to reported past PCR positivity in each survey sample
5. Table. COVID-19-like symptoms, December 2020
6. Table. SARS-CoV-2 Seroprevalence by characteristics of HCW participants in each survey sample
7. Table. Self-reported COVID-19 exposures and infection prevention and control (IPC) measures, May 2020
8. References

**1. Laboratory procedures**

Serological analyses for SARS-CoV-2 were performed using a four-tiered testing strategy (Figure S1). First, all samples were screened with the semiquantitative Euroimmun SARS-CoV-2 IgG antibody ELISA with S1 domain substrate (Euroimmun AG, Lübeck, Germany). The test was carried out according to manufacturer’s instructions. However, single values were analysed and the following adapted cut-offs were applied: samples with ratios <0.5 were considered negative, while samples with ratios ≥0.5 were further evaluated (including ≥0.5-3.0 as borderline and ≥3 as positive). For further evaluation, the Euroimmun Immunoglobulin G (IgG) ELISA was repeated in duplicate and the same cut-offs were applied. For those samples that were repeated in duplicate, mean ratios from the repeat testing were reported. Samples with mean ratios ≥0.5 were further evaluated using the WANTAI SARS-CoV-2 Ab ELISA (Beijing Wantai Biological Pharmacy Enterprise; Beijing, China) as a verification assay according to manufacturer’s instructions with the exception that 50 µl of serum were used. In this case, the following manufacturer-recommended cutoffs were used: <0.9 (negative); ratio ≥ 0.9 – 1.1 (borderline); ≥ 1.1 (positive). When both ELISA results matched, samples were considered positive or negative. In case of borderline results in the Euroimmune ELISA, the result from the Wantai ELISA was used.

If results did not match between Euroimmune and Wantai ELISAs or borderline results were found with the final Wantai ELISA, an in-house biological neutralization test (NT) was performed for the final determination. Briefly, 100 µL serum was diluted in DMEM (10 % FCS, 2 mM L-glutamine) in six two-fold dilutions resulting in dilutions of 1:10 up to 1:320. Dilutions were mixed 1:1 with SARS-CoV-2 (strain BetaCoV/Germany/BavPat1/2020, Institute for Microbiology of the German Armed Forces; final virus concentration 1,000 TCID50 /mL) and incubated at room temperature for 1 h. Next, 100 µL of diluted serum-virus mix were added to wells containing 2 x 10^4^ Vero E6 cells per well (#85020206, European Collection of Authenticated Cell Cultures (ECACC), Porton Down, UK), in a 96-well plate. Each sample dilution was tested in eight replicates and cells were incubated for 5 days at 37 °C, 5 % CO2. After 5 days each well was analysed by light microscopy for visible cytopathic effect (CPE). The number of wells without CPE (negative wells) was counted and PRNT50 values were calculated according to Reed and Muench [1]. For quality control, a positive control with defined titre was analysed in parallel and back-titration of the virus stock was performed. Samples with a titre ≥ 1:15 were considered positive.

**2. Figure. Four-tiered testing strategy for the detection of SARS-CoV-2 antibodies**

**
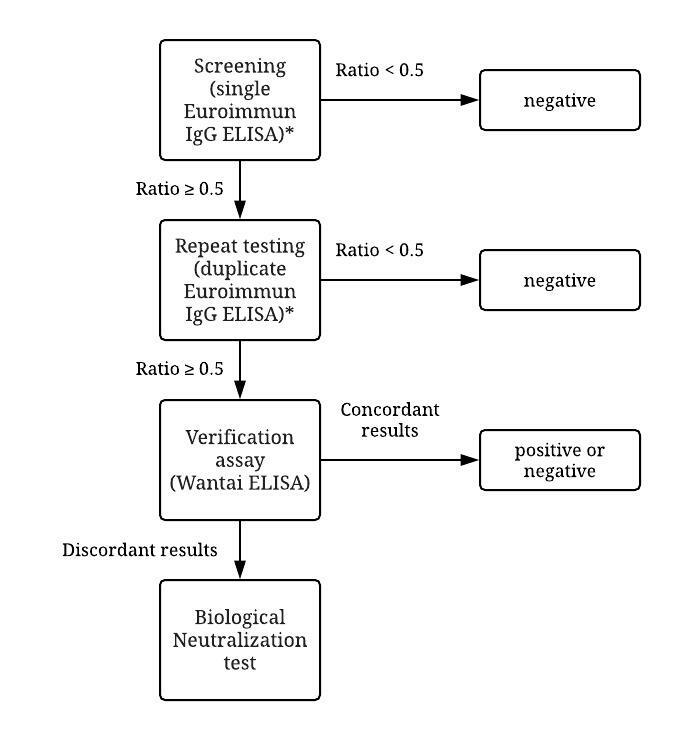
**

*Lots E200414BG and E200423AF in May/June 2020 and Lot E201009AQ in December

**3. Table. Stratified seroprevalence sensitivity analysis of results in December 2020**

| **Estimates** | **Seropositivity (95% CI)** |
| --- | --- |
| Final results according to four-tier testing strategy | 4.58% (3.48%, 5.90%) |
| Results according to Euroimmun ELISA ratios only adjusted for test sensitivity and specificity* | 4.60% (3.34%, 6.11%) |

*Using the function epi.prev in the R package ‘epiR, version 2.0.19, the manufacturer-provided cut-off for seropositivity (ratio ≥1.1) and estimates of sensitivity (88.3%) and specificity (99.2%) only available for the testing lot used in December 2020 from Paul Ehrlich Institute, Germany as previously described [2, 3].

**4. Table. Results of SARS-CoV-2 seropositivity compared to reported past PCR positivity in each survey sample**

| **Reported past PCR positive test result** | **May/June 2020 (N=1477)** | | **December 2020 (N=1223)** | | **Longitudinal sample (N=756)** | | | |
| --- | --- | --- | --- | --- | --- | --- | --- | --- |
|  |  |  |  |  | **May/June 2020** | | **December 2020** | |
|  | **Seropositive**  **(n=18)** | **Seronegative**  **(n=1459)** | **Seropositive**  **(n=56)** | **Seronegative**  **(n=1167)** | **Seropositive**  **(n=7)** | **Seronegative**  **(n=749)** | **Seropositive**  **(n=35)** | **Seronegative**  **(n=721)** |
| Yes | 8 (44.4%) | 4 (0.3%) | 48 (85.7%) | 18 (1.5%) | 3 (42.9%) | 3 (0.4%) | 30 (85.7%) | 13 (1.8%) |
| No/Unknown | 10 (55.6%) | 1455 (99.7%) | 8 (14.3%) | 1149 (98.5%) | 4 (57.1%) | 746 (99.6%) | 5 (14.3%) | 708 (98.2%) |

**5. Table. COVID-19-like symptoms, December 2020 (N=1223)**

| **Symptom** | **Seropositive (N=56)**  **n (%)** | **Seronegative (N=1167)**  **n (%)** |
| --- | --- | --- |
| Cough | 11 (19.6%) | 43 (3.7%) |
| Rhinorrhoea | 11 (19.6%) | 87 (7.5%) |
| Loss of smell/taste | 8 (14.3%) | 8 (0.7%) |
| Body aches | 7 (12.5%) | 27 (2.3%) |
| Sore throat | 4 (7.1%) | 83 (7.1%) |
| Shortness of breath | 3 (5.4%) | 5 (0.4%) |
| Nausea/vomiting/diarrhoea | 2 (3.6%) | 27 (2.3%) |
| Fever > 38° Celsius | 2 (3.6%) | 4 (0.3%) |
| Other symptoms | 2 (3.6%) | 5 (0.4%) |
| Unknown | 1 (1.8%) | 16 (1.4%) |

**6. Table. SARS-CoV-2 Seroprevalence by characteristics of HCW participants in each survey sample**

| **Variable** | **May/June 2020 (n=1477)** | **December 2020 (n=1223)** | **Longitudinal sample (n=756)** | | | |
| --- | --- | --- | --- | --- | --- | --- |
|  |  |  | **May/June 2020** | | **December 2020** | |
| Overall seropositivity |  |  | |  | |  |
| Positive | 18 (1.2%) | 56 (4.6%) | | 7 (0.9%) | | 35 (4.6%) |
| Negative | 1459 (98.8%) | 1167 (95.4%) | | 749 (99.1%) | | 721 (95.4%) |
| Age group, years |  |  | |  | |  |
| 16-29 | 5/189 (2.6%) | 12/176 (6.8%) | | 1/66 (1.5%) | | 4/66 (6.1%) |
| 30-39 | 4/503 (0.8%) | 15/405 (3.7%) | | 1/246 (0.4%) | | 12/246 (4.9%) |
| 40-49 | 5/349 (1.4%) | 20/284 (7.0%) | | 4/195 (2.1%) | | 15/195 (7.7%) |
| 50-59 | 3/358 (0.8%) | 6/281 (2.1%) | | 1/211 (0.5%9 | | 3/211 (1.4%) |
| 60+ | 1/73 (1.4%) | 2/58 (3.4%) | | 0/38 | | 1/38 (2.6%) |
| Unknown | 0/5 | 1/19 (5.3%) | | 0 | | 0 |
| Gender |  |  | |  | |  |
| Female | 12/1038 (1.2%) | 31/842 (3.7%) | | 3/527 (0.6%) | | 16/527 (3.0%) |
| Male | 6/436 (1.4%) | 24/380 (6.3%) | | 4/229 (1.7%) | | 19/229 (8.3%) |
| Diverse | 0/1 | 1/1 (100%) | | 0 | | 0 |
| Unknown | 0/2 | 0 | | 0 | | 0 |
| Self-reported COVID-19-like  symptoms (last 14 days)* |  |  | |  | |  |
| Yes | 7/140 (5.0%) | 21/193 (10.9%) | | 4/68 (5.9%) | | 12/115 (10.4%) |
| No | 10/1148 (0.9%) | 34/1013 (3.4%) | | 3/582 (0.5%) | | 22/627 (3.5%) |
| Unknown | 1/189 (0.5%) | 1/17 (5.9%) | | 0/106 | | 1/14 (7.1%) |
| Type of profession |  |  | |  | |  |
| Nurse | 6/469 (1.3%) | 22/327 (6.7%) | | 2/189 (1.1%) | | 11/189 (5.8%) |
| Physician | 8/307 (2.6%) | 14/264 (5.3%) | | 4/167 (2.4%) | | 10/167 (6.0%) |
| Other allied health professionals | 2/298 (0.7%) | 10/276 (3.6%) | | 1/178 (0.6%) | | 7/178 (3.9%) |
| Administration/Facility management | 1/367 (0.3%) | 9/325 (2.8%) | | 0/211 | | 6/211 (2.8%) |
| Unknown | 1/37 (1.2%) | 1/31 (3.2%) | | 0/11 | | 1/11 (9.1%) |
| Exposition SARS-CoV-2 |  |  | |  | |  |
| High risk | 3/74 (4.1%) | 13/58 (22.4%) | | 1/37 (2.7%) | | 9/37 (24.3%) |
| Moderate risk | 8/204 (3.9%) | 12/200 (6.0%) | | 5/99 (5.1%) | | 10/99 (10.1%) |
| Low risk | 7/831 (0.8%) | 26/619 (4.2%) | | 1/394 (0.3%) | | 13/394 (3.3%) |
| Very low risk | 0/349 | 5/332 (1.5%) | | 0/218 | | 3/218 (1.4%) |
| Unknown | 0/19 | 0/14 | | 0/8 | | 0/8 |

*In May/June, HCWs reported symptoms in the past month; In December, HCWs reported symptoms in the last 14 days

**7. Table. Self-reported COVID-19 exposures and infection prevention and control (IPC) measures**, **May 2020 (N=391)**

| **Question on all HCW participants** | **Seropositive**  **(N=12), n (%)** | | | | **Seronegative**  **(N=379), n (%)** | | | | |
| --- | --- | --- | --- | --- | --- | --- | --- | --- | --- |
| Did you have close contact to a confirmed COVID-19 case outside of the hospital in the last 14 days? |  | | | |  | | | | |
| Yes, with medical mask | 0 | | | | 15 (4.0%) | | | | |
| Yes, with non-medical (fabric) mask | 1 (8.3%) | | | | 1 (0.3%) | | | | |
| Yes, unprotected | 1 (8.3%) | | | | 6 (1.6%) | | | | |
| No | 8 (66.7%) | | | | 319 (84.2%) | | | | |
| I don’t know | 2 (16.7%) | | | | 38 (10.0%) | | | | |
| On how many days did you have close contact to colleagues without a medical mask in the last 14 days? |  | | | |  | | | | |
| 0 | 4 (33.3%) | | | | 110 (29.0%) | | | | |
| 1-4 | 2 (16.7%) | | | | 94 (24.8%) | | | | |
| 5-8 | 5 (41.7%) | | | | 76 (20.1%) | | | | |
| 9-12 | 0 | | | | 50 (13.2%) | | | | |
| 13-14 | 1 (8.3%) | | | | 44 (11.6%) | | | | |
| Did you treat COVID-19 patients in the last 14 days?* |  | | | |  | | | | |
| Yes, confirmed case | 6 (50.0%) | | | | 143 (37.7%) | | | | |
| Yes, suspected case | 6 (50.0%) | | | | 150 (39.6%) | | | | |
| Yes, unknown at the time but patient later tested positive | 1 (8.3%) | | | | 74 (19.5%) | | | | |
| No | 5 (41.7%) | | | | 156 (41.2%) | | | | |
| I don’t know | 0 | | | | 40 (10.6%) | | | | |
| **If yes, you have treated COVID-19 patients in the last 14 days:** | **Seropositive**  **(N=7), n (%)** | | | | **Seronegative**  **(N=183), n (%)** | | | | |
| On how many days did you have contact to a confirmed COVID-19 case (distance <1.5m) in the last 14 days? |  | | | |  | | | | |
| 1-4 | 5 (71.4%) | | | | 79 (43.2%) | | | | |
| 5-8 | 2 (28.6%) | | | | 20 (10.9%) | | | | |
| 9-12 | 0 | | | | 34 (18.6%) | | | | |
| 13-14 | 0 | | | | 12 (6.6%) | | | | |
| I don’t know | 0 | | | | 38 (20.8%) | | | | |
| How many times per day did you have contact to confirmed COVID-19 case within in the last 14 days (distance <1.5m) |  | | | |  | | | | |
| Less than 5 times | 6 (85.7%) | | | | 89 (48.6%) | | | | |
| 5-10 times | 0 | | | | 44 (24.0%) | | | | |
| More than 10 times | 1 (14.3%) | | | | 25 (13.7%) | | | | |
| I don’t know | 0 | | | | 24 (13.1%) | | | | |
| How long was your contact in average (distance <1.5m)? |  | | | |  | | | | |
| Shorter than 5 minutes | 1 (14.3%) | | | | 37 (20.2%) | | | | |
| 5-15 minutes | 4 (57.1%) | | | | 76 (41.5%) | | | | |
| Longer than 15 minutes | 2 (28.6%) | | | | 48 (26.2%) | | | | |
| I don’t know | 0 | | | | 20 (10.9%) | | | | |
| **If yes, you were present for any aerosol generating procedures with (suspected) performed on COVID-19 cases, how often did you…** | **Seropositive**  **(N=4), n (%)** | | | | **Seronegative**  **(N=171), n (%)** | | | | |
|  | **90-100% of the time** | **50-90% of the time** | **20-50% of the time** | **<20%**  **of the time** | **90-100% of the**  **time** | **50-90%**  **of the**  **time** | **20-50% of the time** | | **<20%**  **of the time** |
| wear a medical mask** | 3 (75%) | 1 (25%) | 0 | 0 | 112 (65%) | 5 (3%) | | 2 (1%) | 14 (8%) |
| wear a FFP2/FFP3 respirator | 3 (75%) | 1 (25%) | 0 | 0 | 127 (74%) | 29 (17%) | | 2 (1%) | 10 (6%) |
| put on disposable gloves | 4 (100%) | 0 | 0 | 0 | 160 (94%) | 6 (4%) | | 0 | 2 (1%) |
| put on protective glasses | 3 (75%) | 1 (25%) | 0 | 0 | 123 (72%) | 27 (16%) | | 6 (4%) | 9 (5%) |
| put on protective gown | 4 (100%) | 0 | 0 | 0 | 133 (78%) | 26 (15%) | | 5 (3%) | 4 (2%) |
| perform hand hygiene beforehand | 4 (100%) | 0 | 0 | 0 | 147 (86%) | 15 (9%) | | 5 (3%) | 3 (2%) |
| perform hand hygiene afterwards | 4 (100%) | 0 | 0 | 0 | 166 (97%) | 3 (2%) | | 0 | 2 (1%) |
| **If yes, you were present for other non-aerosol generating procedures with (suspected) performed on COVID-19 cases, how often did you…** | **Seropositive**  **(N=6), n (%)** | | | | **Seronegative**  **(N=177), n (%)** | | | | |
|  | **90-100% of the time** | **50-90% of the time** | **20-50% of the time** | **<20%**  **of the time** | **90-100% of the**  **time** | **50-90%**  **of the**  **time** | | **20-50% of the time** | **<20%**  **of the time** |
| wear a medical mask** | 5 (83%) | 0 | 1 (17%) | 0 | 125 (71%) | 4 (2%) | | 1 (1%) | 15 (9%) |
| wear a FFP2/FFP2 respirator | 4 (67%) | 0 | 1 (17%) | 1 (17%) | 120 (68%) | 25 (14%) | | 7 (4%) | 20 (11%) |
| put on disposable gloves | 5 (83%) | 0 | 0 | 1 (17%) | 162 (92%) | 9 (5%) | | 0 | 6 (3%) |
| put on protective glasses | 3 (50%) | 1 (17%) | 1 (17%) | 1 (17%) | 117 (66%) | 28 (16%) | | 7 (4%) | 21 (12%) |
| put on protective gown | 4 (67%) | 0 | 0 | 2 (33%) | 130 (73%) | 23 (13%) | | 8 (5%) | 14 (8%) |
| perform hand hygiene beforehand | 5 (83%) | 0 | 0 | 1 (17%) | 149 (84%) | 13 (7%) | | 5 (3%) | 7 (4%) |
| perform hand hygiene afterwards | 5 (83%) |  |  | 1 (17%) | 168 (95%) | 2 (1%) | |  | 6 (3%) |
| **Question on all HCW participants** | **Seropositive**  **(N=12), n (%)** | | | | **Seronegative**  **(N=379), n (%)** | | | | |
| Did you have access to a practical IPC training in the context of the COVID-19 outbreak (e.g. donning and doffing of PPE)? |  | | | |  | | | | |
| Yes | 4 (33.3%) | | | | 106 (28.0%) | | | | |
| No | 4 (33.3%) | | | | 51 (13.5%) | | | | |
| I do not know | 4 (33.3%) | | | | 222 (58.6%) | | | | |
| Do you have access to a better fitting mask when necessary? |  | | | |  | | | | |
| Yes | 3 (25.0%) | | | | 103 (27.2%) | | | | |
| No | 5 (41.7%) | | | | 159 (42.0%) | | | | |
| Not applicable | 1 (8.3%) | | | | 73 (19.3%) | | | | |
| I don’t know | 3 (25.0%) | | | | 44 (11.6%) | | | | |
| Have you undergone respirator fit-testing (FFP2/FFP3)? |  | | | |  | | | | |
| Yes | 8 (66.7%) | | | | 148 (39.1%) | | | | |
| No | 2 (16.7%) | | | | 108 (28.5%) | | | | |
| Not applicable | 2 (16.7%) | | | | 105 (27.7%) | | | | |
| I don’t know | 0 | | | | 18 (4.7%) | | | | |

*Respondent could select more than one answer

**>10% of these responses were missing

**8. References**

1. Reed, L.J. and H. Muench, *A SIMPLE METHOD OF ESTIMATING FIFTY PER CENT ENDPOINTS12.* American Journal of Epidemiology, 1938. **27**(3): p. 493-497.

2. Santos-Hövener, C., et al., *Serology- and PCR-based cumulative incidence of SARS-CoV-2 infection in adults in a successfully contained early hotspot (CoMoLo study), Germany, May to June 2020.* Euro surveillance : bulletin Europeen sur les maladies transmissibles = European communicable disease bulletin, 2020. **25**(47): p. 2001752.

3. Steven, M.S., E. *Package ‘epiR’ Version 2.0.19: Tools for the Analysis of Epidemiological Data*. 2021; Available from: <https://cran.r-project.org/web/packages/epiR/epiR.pdf>.
